# Assessment of Micronutrient Deficiencies in Exclusively Breastfed Infants: A Cross-Sectional Study

**DOI:** 10.3390/children12121702

**Published:** 2025-12-17

**Authors:** Burçe Emine Dörtkardeşler, Merve Tosyali, Feyza Koç, Oya Baltalı Hıdır, Güneş Ak

**Affiliations:** 1 Division of General Pediatrics, Department of Pediatrics, Faculty of Medicine, Ege University, 35100 İzmir, Turkey; burce.dortkardesler@ege.edu.tr; 2 Division of Social Pediatrics, Department of Pediatrics, Faculty of Medicine, Ege University, 35100 İzmir, Turkey; feyza.koc@ege.edu.tr; 3 Department of Pediatrics, University of Health Sciences, Tepecik Training and Research Hospital, 35020 İzmir, Turkey; oyahalicioglu@gmail.com; 4 Department of Clinical Biochemistry, Faculty of Medicine, Ege University, 35100 İzmir, Turkey; gunes.ak@ege.edu.tr

**Keywords:** micronutrient deficiency, infant, breastfeeding, vitamin D, iron

## Abstract

**Highlights:**

**What are the main findings?**

• Iron deficiency (40.2%) and vitamin D deficiency (14.4%) were common among exclusively breastfed infants despite national supplementation programs.
• Subclinical vitamin B12 deficiency (29.5%) was frequently observed even in the absence of clinical symptoms or anemia.

**What are the implications of the main findings?**

• Routine supplementation practices should be strengthened and parental adherence closely monitored to reduce preventable deficiencies.
• Early evaluation of maternal and infant B12 status should be considered in developing countries to identify and manage subclinical deficiencies.

**Abstract:**

**Background/Objectives**: Micronutrient deficiencies during infancy remain a major public health concern, particularly in developing countries. Although exclusive breastfeeding is the optimal source of nutrition for infants up to six months of age, limited studies have simultaneously evaluated multiple micronutrient statuses in this population. This study aimed to assess the levels of vitamin D, iron, and other micronutrients—including vitamins A, E, B12, folic acid, zinc, and copper—in exclusively breastfed infants. **Methods**: This cross-sectional study was conducted between 2022 and 2024 at a university well-child clinic in İzmir, Turkey. A total of 132 healthy, exclusively breastfed six-month-old infants were included. Blood samples were analyzed for hemogram, serum iron, ferritin, 25(OH)D, vitamins A, E, B12, folic acid, zinc, and copper. Sociodemographic data and supplementation practices were recorded. Deficiency cut-offs were defined according to WHO and national guidelines. **Results**: Among the infants, 40.2% had iron deficiency or anemia, and 14.4% had vitamin D deficiency. Vitamin B12, A, E, zinc, and copper deficiencies were identified in 29.5%, 2.4%, 4%, 7.4%, and 6.6% of infants, respectively. Regular vitamin D and iron supplementation were significantly associated with lower deficiency rates (*p* < 0.05). Maternal education level, dressing style, and smoking status were significantly related to infant vitamin D status. **Conclusions**: Despite national supplementation programs, iron and vitamin D deficiencies remain common in exclusively breastfed infants. Routine and regular supplementation should be emphasized, and subclinical deficiencies—particularly vitamin B12—should be considered. Broader studies evaluating maternal nutritional factors and socioeconomic determinants are warranted to guide preventive strategies.

## 1. Introduction

Micronutrients are vitamins and minerals that are essential for the physiological functions of the human body. Micronutrient deficiencies (vitamin D, A, iron, iodine, zinc, etc.) are a global health problem. It is estimated that more than 2 billion people worldwide have vitamin and mineral deficiencies [1]. People in low- and middle-income countries, pregnant women, breastfeeding women, and young children are particularly at risk. Micronutrient deficiencies can cause significant symptoms, including loss of appetite, increased susceptibility to infections, anemia, neurological problems, visual impairment, and delays in growth and development.

To obtain the appropriate amounts of micronutrients, which are essential for growth and development in children, it is necessary to use food sources effectively. The World Health Organization (WHO) recommends exclusive breastfeeding for the first 6 months and then continued breastfeeding until at least 2 years of age, with the addition of complementary foods [2]. It was reported that the micronutrients in the breastmilk of a healthy mother who did not use a restricted diet (vegetarian diet vs) were sufficient for infants until the end of the 6th month [1,3,4,5]. However, complementary foods should be started at the end of the 6th month to provide energy, vitamins, and minerals [2,6].

Supplementation of vitamin D and iron is recommended for infants who are exclusively breastfed [7,8,9,10,11,12]. In our country, the supplementation of vitamin D has been provided free of charge to infants from birth up to the age of 1, as recommended by the Ministry of Health, since 2005 [13]. Similarly, iron prophylaxis is recommended for term infants from the fourth month of life until at least one year of age [14]. However, despite the use of vitamin D and iron supplements, deficiency of these micronutrients continued to be found in our country [15,16]. It is also known that there are deficiencies in other micronutrients [4,17,18].

There have not been many studies that evaluated micronutrients together, especially in healthy infants and early childhood [5]. The studies conducted often concern iron and/or vitamin D, while studies on other micronutrients have been conducted in older children [19,20,21]. This study aimed to determine the levels of vitamin D, iron, and affecting factors, as well as micronutrient levels such as vitamins A, E, B12, folic acid, zinc, and copper in healthy infants who were exclusively breastfed during early infancy. As a result of this study, we aimed to provide knowledge about micronutrient deficiency in exclusively breastfed infants.

## 2. Materials and Methods

### 2.1. Study Design

This study was conducted between January 2022 and 2024 at Medical School Well-Child Care Clinics in Izmir, Turkey. This study was conducted by the principles outlined in the Declaration of Helsinki, 2008, and approved by the local ethics committee (No.: 11-9.1/4-19 October 2011). Informed consent was obtained from all parents. A total of 150 children were included in the study. The study population consisted of healthy infants who were 6 months old, exclusively breastfed, and given vitamin D (400–800 IU/day) immediately after birth, as well as iron prophylaxis (1 mg/kg/day) at 4 months, and continued to use it. Infants with a history of premature birth, intrauterine growth retardation, a previously known chronic disease, growth and developmental retardation, acute infection, infants who were not breastfed, received formula or supplementary food other than breast milk received micronutrient supplements other than iron and vitamin D were excluded from the study.

A questionnaire was used, including the infants’ weight, height, gender, and sociodemographic characteristics of the family (economic status, education, and employment status), infants’ vitamin D and iron usage status (regular usage), mothers’ number of births, nutritional history, dressing style, smoking status and mothers’ multivitamin supplementation during pregnancy.

Blood samples were taken from the infants for hemogram, serum iron, ferritin, 25 (OH) vitamin D, vitamin A (serum retinol), vitamin E (alpha-tocopherol), vitamin B12, folic acid, zinc and copper levels. Dates of blood sampling were recorded.

### 2.2. Sample Collection and Laboratory Analysis

Venous blood samples (~4 mL) were collected from infants in accordance with standard blood collection guidelines and immediately centrifuged at 4000 rpm for 10 min. All analyses were performed on the same day in the clinical biochemistry laboratory. Hemogram, iron, ferritin, and other micronutrient levels were evaluated. Serum vitamin B12 and folic acid levels were measured using the electrochemiluminescence method (Cobas^®^ 8000, Roche Diagnostics, Mannheim, Germany) (normal values: vitamin B12, 197–866 pg/mL, folic acid, 3.89–26.8 ng/mL). Serum vitamin A and E levels were quantified by high-performance liquid chromatography (HPLC) (LC-20AT, Shimadzu^®^, Kyoto, Japan) (normal values: vitamin A, 300–800 µg/L, vitamin E, 6.6–14.3 mg/L). Serum 25-hydroxyvitamin D (25-OH vitamin D) levels were determined via liquid chromatography-mass spectrometry (LC-MS) (LCMS-8040, Shimadzu^®^, Kyoto, Japan) (normal values: 20–50 ng/mL). Serum zinc and copper concentrations were analyzed using atomic absorption spectrophotometry (AAS) (AA-7000, Shimadzu^®^, Kyoto, Japan) (normal values: zinc, 51–120 µg/dL, copper, 13–140 µg/dL).

### 2.3. Definitions and Descriptions

When the dressing characteristics of the mothers were evaluated, ‘covered’ was defined as covering the head and arms and not covering the hands and face with clothing, while ‘uncovered’ was defined as leaving the head and arms. When mothers were asked about their frequency of dairy product consumption, consuming at least 200 mL of milk or equivalent dairy products such as yogurt and cheese every day or 4–5 days a week was considered “sufficient” dairy product consumption, while consuming dairy products 3 times a week or less was considered “insufficient” [22]. Red meat consumption more than 1–2 days a week was defined as “sufficient” [22]. The parents were asked, “How do you perceive your economic situation?” The answers were recorded as medium, rich, and poor.

Vitamin D and iron use were questioned. Daily use was defined as “regular use”, while using 6 times or less per week was defined as “irregular use”.

According to the dates of blood sampling, summer season was defined as 1 May to 30 September, and winter season was defined as 1 October to 30 April, according to the latitude of the geographic region of the study.

When the iron status of the children included in the study was evaluated; Serum iron (SI) < 30 mg/dL and/or Ferritin < 12 µg/L was defined as iron deficiency (ID) and SI < 30 mg/dL and/or Ferritin < 12 µg/L, Hemoglobin (Hb) < 11 g/dL was defined as iron deficiency anemia (IDA) [23,24]. A 25(OH)D level of <20 ng/mL was defined as deficiency, and a level of ≥20 ng/mL was defined as normal [25]. Vitamin B12 levels <200 pg/mL and folic acid levels <4 ng/mL were considered as deficiencies [26,27,28]. Serum vitamin A (serum retinol) < 200 μg/L, Vitamin E (alpha-tocopherol) < 5 mg/L, zinc < 65 μg/dL, and copper < 75 μg/dL were evaluated as deficiency [29,30,31,32].

### 2.4. Data Analysis

Descriptive statistics of the data are given as mean, standard deviation, median, minimum, maximum, frequency, and percentage values. The normality assumption of quantitative data was checked with the Shapiro–Wilk test. Although several micronutrient variables were not normally distributed, given the relatively large sample sizes (n > 50 for most biochemical parameters), continuous variables were summarized as mean ± standard deviation to facilitate comparison between groups, while the choice of statistical test was based on the normality assessment. The Independent samples *t*-test was used for variables showing normal distribution, and the Mann–Whitney U test was used for variables that did not meet the normality assumption. Relationships between categorical variables were examined using the Pearson Chi-square test and Fisher’s Exact probability test. Statistical analyses were performed using the IBM SPSS Statistics 25.0 (IBM SPSS Statistics for Windows, Version 25.0. Armonk, NY, USA: IBM Corp.) package program. The significance level was determined as 0.05 in all analyses.

## 3. Results

A total of 150 infants were included in this study. Because 8 of the infants did not meet the inclusion criteria, insufficient blood samples were taken from 6 infants, and the mothers of 4 did not want to participate in the study, the study was performed with 132 infants, of which 59 were female. The sociodemographic characteristics are shown in Table 1. It was determined that 65 (49.2%) of the mothers were university graduates, 68 (51.5%) were employed, and 95 (72%) used multivitamins during pregnancy. The mothers’ nutritional characteristics were found; 90.1% of them had sufficient milk and dairy product consumption, and 72.7% insufficient red meat consumption. It was determined that there were no mothers who preferred a vegetarian diet or a different diet (gluten-free diet, etc.). Ninety-seven per cent of the families perceived their economic situation as medium.

Micronutrient levels and deficiency status of the infants are shown in Table 2. In addition, hemoglobin (Hb) values of the infants were determined as 11.3 ± 0.9 gr/dL, and ferritin as 49.9 ± 35.6 µg/L. The mean iron and vitamin D values of the infants in the study were found to be 55.5 ± 21.5 mg/dL and 38.5 ± 16.9 ng/mL, respectively. It was determined that 40.2% of the infants had ID/IDA, and 14.4% had vitamin D deficiency. When the vitamin D supplementation of the infants was evaluated, it was shown that 96.2% of them used it regularly. It was determined that 87 (65.9%) of the infants were given 400 IU/day while 45 (34.1%) of the infants were given 800 IU/day of vitamin D. It was found that 87.9% (n = 116) of the infants used iron supplements regularly.

In our study, infants were evaluated in terms of factors affecting vitamin D deficiency. (Table 3). No difference was found between the groups with and without vitamin D deficiency in terms of gender, height, weight, economic status, daily dose, mother’s employment, use of multivitamins during pregnancy, milk, and dairy product consumption, and time of blood collection (*p* > 0.05). However, when the mother’s education and dressing style, smoking status, and regular use of vitamin D were evaluated, a statistically significant difference was found between the two groups (*p* < 0.05). There were no clinical signs of rickets in the infants with vitamin D deficiency.

No statistically significant difference was found between the two groups with and without iron deficiency in terms of gender, weight, economic status, mother’s employment, smoking status, use of multivitamins during pregnancy, and red meat consumption (Table 4, *p* > 0.05). It was determined that 64.6% (n = 75) of those who used iron supplements regularly did not have iron deficiency, and 35.4% (n = 41) had iron deficiency (*p* < 0.05).

Vitamin B12, A, E, zinc, and copper deficiencies were found to be 29.5%, 2.4%, 4%, 7.4%, and 6.6%, respectively (Table 2). None of the infants with the deficiency have clinical findings. No statistically significant difference was found between the two groups with and without vitamin B12 deficiency in terms of gender, height, weight, economic status, mother’s employment, smoking status, use of multivitamins during pregnancy, and red meat consumption (*p* > 0.05). Anemia was not detected in infants with B12 deficiency.

## 4. Discussion

In our study, it was determined that 40.2% of infants had iron deficiency (ID) and iron deficiency anemia (IDA), and 14.4% had vitamin D deficiency. Vitamin B12, A, E, zinc, and copper deficiencies were found to be 29.5%, 2.4%, 4%, 7.4%, and 6.6%, respectively. There were not many studies that evaluated micronutrients together in early infancy in healthy infants who are exclusively breastfed. Therefore, our study is important in terms of making a scientific contribution to this field.

Iron deficiency is the most common micronutrient deficiency in children. Previous studies have reported different rates of iron deficiency in childhood, such as 6.5–42% [16]. Özden et al. [33] reported that 51.4% of 6-month-old infants had iron deficiency in their study. In a recent study, 31.9% of 1-year-old infants were found to have ID/IDA [34]. As the need for iron increases after 6 months of age, both iron-rich complementary foods and iron supplementation are globally recommended for predominantly or exclusively breastfed infants. The World Health Organization recommends routine iron supplementation for infants aged 6–23 months in countries where the prevalence of iron-deficiency anemia exceeds 40% [12]. In our country, iron supplementation is also a key component of infant health monitoring. In line with WHO guidance, infants begin routine iron prophylaxis at 4 months of age as part of the national public health program [14]. When groups with and without iron deficiency were compared, it was determined that iron deficiency was less common when regular iron prophylaxis was used (Table 4, *p* = 0.005). The studies have shown that families are hesitant to use iron preparations due to their gastrointestinal side effects, and this causes irregular use [35,36]. Families should be informed about that, and the infant’s usage characteristics should be questioned at each follow-up visit. It is also very important to know the iron status and diet characteristics of breastfeeding mothers to protect them from iron deficiency because the mother’s iron status and nutrition also affect the infant’s iron status [33]. In our study, there were no mothers on a vegetarian diet, and no relationship was found between the mothers’ red meat consumption and the infant’s iron status. However, in our study, the mothers’ detailed nutritional history (iron-rich food content, etc.) and iron status were not evaluated.

Data on vitamin D deficiency varies according to countries and age groups. The study found that 27.9% of exclusively breastfed 4-month-old infants had vitamin D deficiency [15]. In our study, vitamin D deficiency was detected in 14.4%. In addition, similar to other studies, the mother’s low level of education, covered dress, and irregular use of vitamin D were determined as factors that increased the deficiency [10,15,37,38]. Studies have determined that the season of blood sampling affects the infant’s vitamin D level [15,39]. Some studies have also stated that there is no seasonal difference, similar to our study [40,41]. It is known that maternal vitamin D deficiency is an important risk factor, but the vitamin D levels of mothers were not evaluated in our study.

In our study, vitamin D levels were not found to be statistically different between infants receiving 400 IU and 800 IU daily. Recommended daily doses of vitamin D may vary between countries. In Canada, 400 IU/day is recommended in the summer, 800 IU/day in the winter, and 800 IU/day is recommended in Bulgaria [42,43]. In our country, the Ministry of Health recommended daily use of 400 IU up to the age of 1. As determined in our study, it has been reported that 400 IU/day of vitamin D is sufficient in our country [44].

As an important result of our study, B12 deficiency was detected in 29.5% of healthy 6-month-old infants who were exclusively breastfed. No clinical findings or anemia were detected in these infants. Studies have reported that B12 deficiency in children can be seen as 7.7–41% [45]. In a study conducted on infants and children aged 6–59 months, B12 deficiency was found to be 22.5% [46]. B12 deficiency in early infancy is often related to the mother’s diet and B12 level [18,47]. In our study, there were no mothers on a vegan diet; it was determined that 72.7% of the mothers had insufficient red meat consumption, but no difference was found between the groups with and without B12 deficiency. The mothers’ diet was not questioned concerning B12 sources other than meat products, and the mothers’ B12 levels were not evaluated. Screening for B12 deficiency is not performed in routine child health monitoring. In underdeveloped and developing countries, even if there are no symptoms (with the mother being evaluated), B12 levels may be considered. However, studies evaluating all the factors that may be effective in a larger number of infants in our country need to be conducted.

Subclinical vitamin A deficiency is quite common in the world. The World Health Organization recommends routine supplementation for infants and children between 6–59 months of age in countries where vitamin A deficiency is common [48]. However, in studies conducted in our country, a serious deficiency for vitamin A has been determined at low rates (0.2%), and marginal deficiency has been reported as 16–22% [49,50]. In our study, 2.4% marginal deficiency (vitamin A = 100–200 µg/L) was determined, and there were no cases with serious deficiency.

When other micronutrients were evaluated, vitamin E, zinc, and copper levels were determined as 12.8 ± 9.7 mg/L, 99 ± 26.9 µg/dL, and 104 ± 26.4 µg/dL, respectively. It is difficult to determine the limit values of these micronutrients in healthy children. In our study, the limit values determined for childhood were taken into consideration. Subclinical micronutrient deficiencies may occur in early childhood, although not at very high rates, and should be evaluated together with clinical findings. Studies have shown that micronutrients (especially zinc and copper levels), which are particularly found to be subclinically deficient, are related to the mother’s level and may increase in the following months (without treatment) [33,51].

Beginning at 6 months of age, breast milk alone is no longer sufficient to meet the increasing nutritional requirements of growing infants. Therefore, timely and appropriate complementary feeding becomes essential, particularly for infants who will continue to breastfeed until two years of age. Our findings of subclinical deficiencies—most notably in iron, vitamin D, and vitamin B12—even among exclusively breastfed, otherwise healthy infants highlight the importance of introducing nutrient-dense complementary foods at the recommended age. These deficiencies may represent early warning signs indicating that, without adequate complementary dietary sources, infants are at risk of progressing to more severe micronutrient insufficiencies. Ensuring the quality, diversity, and micronutrient density of complementary foods is crucial to support optimal growth and neurodevelopment during this vulnerable period.

### Limitations

Our study was conducted in a single center, and the number of participants in the defined subgroups was relatively small. Furthermore, considering the high educational attainment of the parents and the reported household income levels, the study sample likely represents a more socioeconomically advantaged population. Therefore, the findings may not be generalizable to the broader population.

Both family income and maternal education are known to influence exclusive breastfeeding (EBF) practices. However, these factors become even more critical when interpreting infant micronutrient levels, as these are closely associated with the mother’s nutritional status, dietary intake, and micronutrient levels. In the present study, we did not measure maternal micronutrient levels, and household income was based on self-report.

There is a need for future studies that classify income levels using objective national criteria, ensure balanced distribution of participants across income groups, and include concurrent assessments of maternal nutritional status and micronutrient levels. Such studies would provide more comprehensive insights into the socioeconomic and maternal determinants of infant micronutrient deficiencies.

In addition, future prospective cohort studies are needed to monitor infants’ micronutrient status over time and determine whether subclinical deficiencies normalize or worsen. Such studies would allow for a clearer understanding of how changes in micronutrient levels relate to growth outcomes—including stunting, undernutrition, and later overweight or obesity—as well as cognitive and neurodevelopmental indicators. Long-term follow-up designs would provide more robust evidence to guide nutritional policies and targeted interventions in early childhood.

## 5. Conclusions

The most appropriate diet for infants is exclusive breastfeeding for the first 6 months, followed by the introduction of complementary foods while continuing breastfeeding until at least 2 years of age. Although breast milk provides many essential micronutrients during early infancy, exclusively breastfed infants still require vitamin D and iron supplementation. Despite routine national use, deficiencies in these micronutrients continue to occur. Therefore, it is essential to ensure regular supplementation and to assess and support mothers’ nutritional status when needed to prevent deficiencies during infancy. Moreover, since various regions remain at risk for multiple micronutrient inadequacies, attention should also be given to preventing other subclinical deficiencies in childhood. Subclinical deficiencies, particularly vitamin B12, may occur even in asymptomatic exclusively breastfed infants, and detailed evaluation may be warranted in populations at higher risk. Comprehensive, multicenter studies that represent different regions and age groups are needed to better understand the determinants of these deficiencies at the national level. Although derived from a single-center cohort, our findings provide valuable evidence that may support future large-scale studies as well as ongoing national efforts to strengthen infant vitamin D and iron supplementation policies.

## Figures and Tables

**Table 1:** Demographic characteristics, (n, %).

	n (%)
Weight (g) *	7999 ± 1056
Length (cm) *	67.2 ± 2.7
Age (days) *	183.39 ± 10.06
Gender	
Male	73 (55.3)
Female	59 (44.7)
Mother’s education	
1–8 years	21 (15.9)
9–12 years	46 (34.8)
University	65 (49.2)
Father’s education	
1–8 years	26 (19.7)
9–12 years	39 (29.5)
University	67 (50.8)
Occupational status of mother	
Housewife	64 (48.5)
Working	68 (51.5)
Family’s financial situation	
Poor	4 (3)
Medium	128 (97)
The mother’s first pregnancy	
Yes	107 (81)
No	25 (19)
Mother’s smoking status	
Yes	17 (12.9)
No	115 (87.1)
Multivitamin supplementation during pregnancy	
Yes	95 (72)
No	37 (28)

* mean ± SD, n = 132.

**Table 2:** Evaluation of micronutrient status of infants.

	Average Laboratory Values (Mean ± SD) *	Deficiency, n (%) *	Normal, n (%) *
Serum Iron (n = 132)	55.5 ± 21.5 mg/dL	53 (40.2)	79 (59.8)
Serum 25(OH) D vit (n = 132)	38.5 ± 16.9 ng/mL	19 (14.4)	113 (85.6)
Serum Vitamin B12, (n = 132)	292.7 ± 175.6 pg/mL	39 (29.5)	93 (70.5)
Serum Folic acid (n = 132)	17 ± 3 ng/mL	0	132 (100)
Serum Vitamin A (n = 124)	358 ± 119 µg/L	3 (2.4)	121 (97.6)
Serum Vitamin E (n = 124)	12.8 ± 9.7 mg/L	5 (4)	119 (96)
Serum Zinc (n = 81)	99 ± 26.9 µg/dL	6 (7.4)	75 (92.6)
Serum Copper (n = 76)	104 ± 26.4 µg/dL	5 (6.6)	71 (93.4)

* Continuous variables are presented as mean ± standard deviation, deficiency status is presented as n (%).

**Table 3:** Factors affecting 25(OH) vitamin D deficiency (n, %).

	Vitamin D Deficiency n = 19	Normal Vitamin D n = 113	*p*
Gender			0.457
Male	12 (63.2)	61 (54)	
Female	7 (36.8)	52 (46)	
Mother’s education			0.015
1–8 years	7 (36.8)	14 (12.4)	
9–12 years	6 (31.6)	40 (35.4)	
University	6 (31.6)	59 (52.2)	
Occupational status of mother			0.17
Housewife	12 (63.2)	52 (46)	
Working	7 (36.8)	61 (54)	
Family’s Financial Situation			0.53
Poor	1 (5.3)	3 (2.7)	
Medium	18 (94.7)	110 (97.3)	
Vitamin D supplementation			0.021
Regular	16 (84.2)	111 (98.2)	
Irregular	3 (15.8)	2 (1.8)	
Daily dose of vitamin D			0.43
400 IU	11 (57.9)	76 (67.3)	
800 IU	8 (42.1)	37 (32.6)	
Mother’s smoking status			0.05
Yes	5 (26.3)	12 (10.6)	
No	14 (73.7)	101 (89.4)	
Multivitamin supplementation during pregnancy			0.46
Yes	15 (78.9)	80 (70.8)	
No	4 (21.1)	33 (29.2)	
Mother’s consumption of dairy products			0.32
Sufficient	14 (73.6)	105 (92.9)	
Insufficient	5 (26.4)	8 (7.1)	
The season of blood sampling			0.85
Summer season	9 (47.4)	51 (45.1)	
Winter season	10 (52.4)	62 (54.9)	
Mothers’ dressing style			0.009
Uncovered	13 (68.4)	102 (90.3)	
Covered	6 (31.6)	11 (9.7)	

**Table 4:** Factors affecting iron deficiency (n, %).

	No ID * n = 79	ID/IDA * n = 53	*p*
Gender			0.35
Male	46 (58.2)	26 (50)	
Female	33 (41.8)	26 (50)	
Mother’s education			0.83
1–8 years	14 (17.7)	7 (13.2)	
9–12 years	29 (36.7)	17 (32)	
University	36 (45.6)	29 (54.7)	
Occupational status of mother			0.66
Housewife	36 (45.6)	28 (52.8)	
Working	43 (54.4)	25 (47.2)	
Family’s Financial Situation			0.74
Poor	3 (3.8)	1 (1.9)	
Medium	76 (96.2)	52 (98.1)	
Iron supplementation			0.005
Regular	75 (94.9)	41 (77.4)	
Irregular	4 (5.1)	12 (22.6)	
Mother’s smoking status			0.50
Yes	9 (11.4)	8 (15.4)	
No	70 (88.6)	45 (84.6)	
Multivitamin supplementation during pregnancy			0.57
Yes	57 (72.1)	38 (71.7)	
No	22 (27.9)	15 (28.3)	
Mother’s consumption of meat			0.97
Sufficient	21 (16.6)	15 (28.3)	
Insufficient	58 (73.4)	38 (71.7)	
The mother’s first pregnancy			0.49
Yes	66 (83.5)	41 (77.3)	
No	13 (16.5)	12 (22.7)	

* ID: Iron Deficiency, IDA: Iron Deficiency Anemia.

## Data Availability

The data presented in this study are available on request from the corresponding author due to ethical reasons.

## Abbreviations

The following abbreviations are used in this manuscript:

EBF	Exclusive Breastfeeding
ID	Iron Deficiency
IDA	Iron Deficiency Anemia
AAS	Atomic Absorption Spectrophotometry
Hb	Hemoglobin
HPLC	High-Performance Liquid Chromatography
LC-MS	Liquid Chromatography-Mass Spectrometry
SI	Serum Iron
WHO	World Health Organization
